# Structure and *m*/*z* of Singly Charged Even-Electron Fragment Ions in Organic Mass Spectrometry: “A Rule of Mass Shift” Revisited (Secondary Publication)

**DOI:** 10.5702/massspectrometry.A0074

**Published:** 2019-11-25

**Authors:** Hisao Nakata

**Affiliations:** 1 Professor Emeritus of Aichi Kyoiku University, 2–68–314 Naruko-cho, Midori-ku, Nagoya 458−0041, Japan

**Keywords:** rule of mass shift, fragmentation, stable fragment ion, prediction of *m*/*z*

## Abstract

Typical modes of bond cleavages of organic compounds in mass spectrometry are briefly summarized. Although these fragmentation rules can be quite useful for interpreting mass spectra of simple compounds, application to structurally complex molecules that contain multiple hetero atoms such as nitrogen or oxygen becomes increasingly difficult, because the exact location of an unpaired electron or positive or negative charges becomes obscure in precursor ions.

About a decade ago, we proposed “a rule of mass shift,” which correctly predicts the *m*/*z* for observed peaks corresponding to singly charged even-electron fragment ions. The basis of the rule postulates that ions observed as peaks in an ordinary mass spectrum should be sufficiently stable to survive during the flight path in a mass spectrometer.

The important recognition is that each atom in a stable ion should be in an ordinary valence state, and no free valence should be allowed. Therefore, if the cleavage of a bond leads to an ion with an unstable structure, some structural changes must take place in order for the ion to be observed in the mass spectrum. Such structural changes can be the addition of hydrogen atom(s) and/or a proton for positive ions, and the addition of a hydrogen atom and/or the elimination of two hydrogen atoms in the case of negative ions. These required structural changes in each case are schematically depicted and discussed in detail.

Two typical examples are shown, in which *m*/*z*’s of the observed peaks are correctly predicted. The scope and limitations, as well as the significance of the rule for analyzing fragmentations in organic mass spectrometry are also discussed.

## 1. INTRODUCTION

Organic mass spectrometry is an analytical method based on the fragmentation of organic ions produced in a mass spectrometer. The questions concerning the mechanism of bond cleavages and the structure of the resultant ions have posed important challenges for understanding mass spectral information. Since the advent of mass spectral measurements for normal organic compounds, this issue has been debated in various ways. Attempts to provide explanation particularly from the standpoint of bond cleavage by “Rules of Fragmentation,” have made great contribution from mid-1960s to 1990, yielding many valuable conclusions and creating a new field of “Chemistry of Organic Ions in the Gas Phase.”

As a generally applicable rule, the method presented in those works is to estimate the position of an unpaired electron or charge, and how they are distributed on the source ion (precursor ion) before fragmentation, and to classify the mode of bond cleavages caused by the unpaired electron or by the charge as a single-electron or two-electron moves depicted respectively as a single-headed arrow (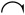) or a double-headed arrow (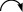). It is no exaggeration to say that those effort at least provides a rational understanding of the fragmentation pattern of most organic compounds in electron ionization (EI) and chemical ionization (CI) mass spectrometry, and elucidates how ions of organic compounds behave in the gas phase.

However, we do not yet have a definite understanding of the relationship between the fragmentation rules and the structure of fragment ions produced as a result of the bond cleavage of organic ions. In normal organic compounds, but not ions, structure and reactivity are closely related to each other. The “structure” of a given molecule determines the “reactivity” of the molecule, and conversely, its “reactivity” can reveal a lot about its “structure.” Therefore, the structure and the reactivity should be regarded as two fundamental and important aspects that reflect unique “characteristic” of organic molecules.^1)^

Surprisingly, however, only little attention is focused on the similar argument for organic ions in the gas phase. The present author has been investigating the relationship between fragmentation and the structure of resultant ions to clarify what structural factor is required for ions observed in the mass spectra, and proposed a “rule of mass shift” that can be applied to the fragmentation of organic ions produced by various new ionization methods.^2,3)^ In this article here, we provide a commentary on the background of how this mass shift rule was established and how this rule has been developed, and discuss the scope and limitations for its applicability.

## 2. RULES FOR BOND CLEAVAGE DURING FRAGMENTATION

Considerable efforts have long been devoted by many research groups to construct fragmentation rules of organic compounds in the gas phase. Before going into detailed discussion of the “rule of mass sift,” the so-called fragmentation rules are briefly summarized first.

During the last couple of decade, the present author has discussed the fragmentation of organic ions in a book,^4)^ several book chapters,^5–7)^ review articles,^8–10)^ lectures and seminars.^11–23)^ By referring to the comprehensive summary by H. Budzikiewicz, C. Djerassi, and D. Williams,^24–26)^ by K. Biemann,^27)^ by F. W. McLafferty^28–30)^ and with F. Tureček,^31)^ by P. Longevialle,^32)^ by Q. N. Porter’s analysis of heterocyclic compounds,^33)^ and by K. Levsen’s physical organic chemistry based discussions,^34)^ the classification of bond cleavages has been systematically improved, and a uniform guide of bond cleavage valid for both positive and negative ions^35–37)^ was presented. Of course, this does not put an end to the issue. The problem should be regarded as a developing one and we discuss it below to make our point clearer.

Since each mode of cleavage is a basic general rule, its application needs to be applied flexibly, keeping in mind the various empirical rules that accompany it. There might probably be many anomalies too. In addition to what is described here, it goes without saying that basic knowledge about the reaction of organic ions in ordinary solution chemistry should be maximized while considering the application to reactions in the gas phase.

### 2.1 Change in bonding due to unpaired electrons

Generally, in the fragmentation of odd-electron ions caused either by unpaired electrons or by charges, an unpaired-electron based cleavage is favored (Table 1). Among the types of cleavage modes depicted in Table 1, cleavage A is exceedingly preferred in ions with unpaired electrons and therefore is a very important mode of cleavage. It is empirically known that the ease of cleavage varies depending on the heteroatom Y, where the unpaired electron is localized, and is likely to follow the order N>O>S>Br>Cl. In addition, as Z is cleaved with an unpaired electron, the cleavability is related to its stability as a radical, and is likely to follow the order RO·>HO·>R· (*tert.*>*sec.*>*prim.*)>CH_3_·>H· (where R denotes an alkyl group).

**Table 1:** Table 1. Bond cleavages caused by an unpaired electron.

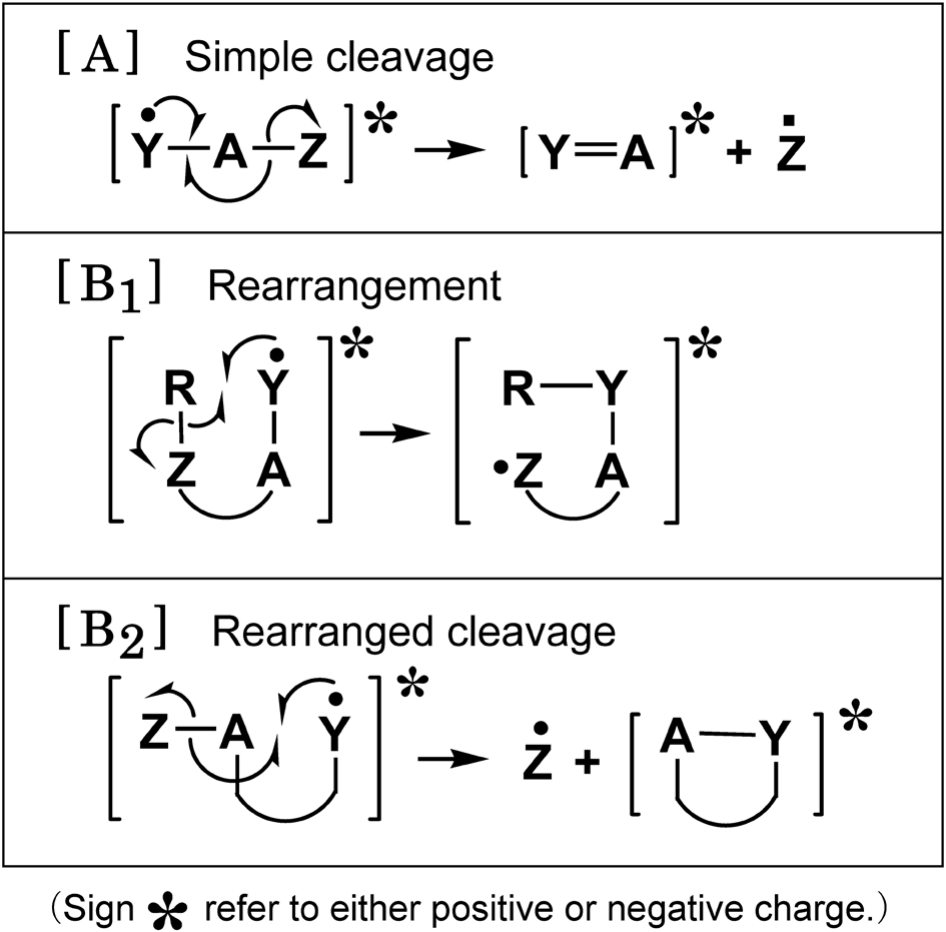

On the other hand, the rearrangement reaction of B_1_, also caused by unpaired electrons, effects no change in the nominal mass of the ion, as only the atom group R changes its bonding position within the ion. As a result, the initial and final ions of the reaction cannot be distinguished from the spectrum. However, the aforementioned type A cleavage follows immediately afterwards, with the help of the unpaired electron now placed on Z, thereby splitting the whole ion into two fragments. This rearrangement reaction is, in most cases, a hydrogen rearrangement (R=H) proceeding through a six-membered ring transition state, as typified to the McLafferty cleavage in carbonyl compounds. Depending on the situation, the rearrangement may even proceed through a five- or four-membered ring transition state. The ease of rearranging hydrogen is related to the stability of the unpaired electron on atom Z after the rearrangement and in the order of >CH->-CH_2_->-CH_3_>=CH-.

In contrast to B_1_, the reaction of B_2_ involves a cleavage of an ion into two fragments. It has been shown that the major difference in ions preferring B_2_ cleavage than B_1_ is that Z is an alkyl group in most cases and that B_2_ is preferred when a five-membered ring transition state is likely to be formed.

### 2.2 Change in bonding due to a formal charge

Cleavage caused by charge (Table 2) occurs in most cases from even-electron ions that have only charges and no unpaired electrons. In this situation, it is normal for the generated fragment ion to be also an even-electron ion, and this is known as “Even-electron rule.”^31,34,38)^

**Table 2:** Table 2. Bond cleavages caused by charges.

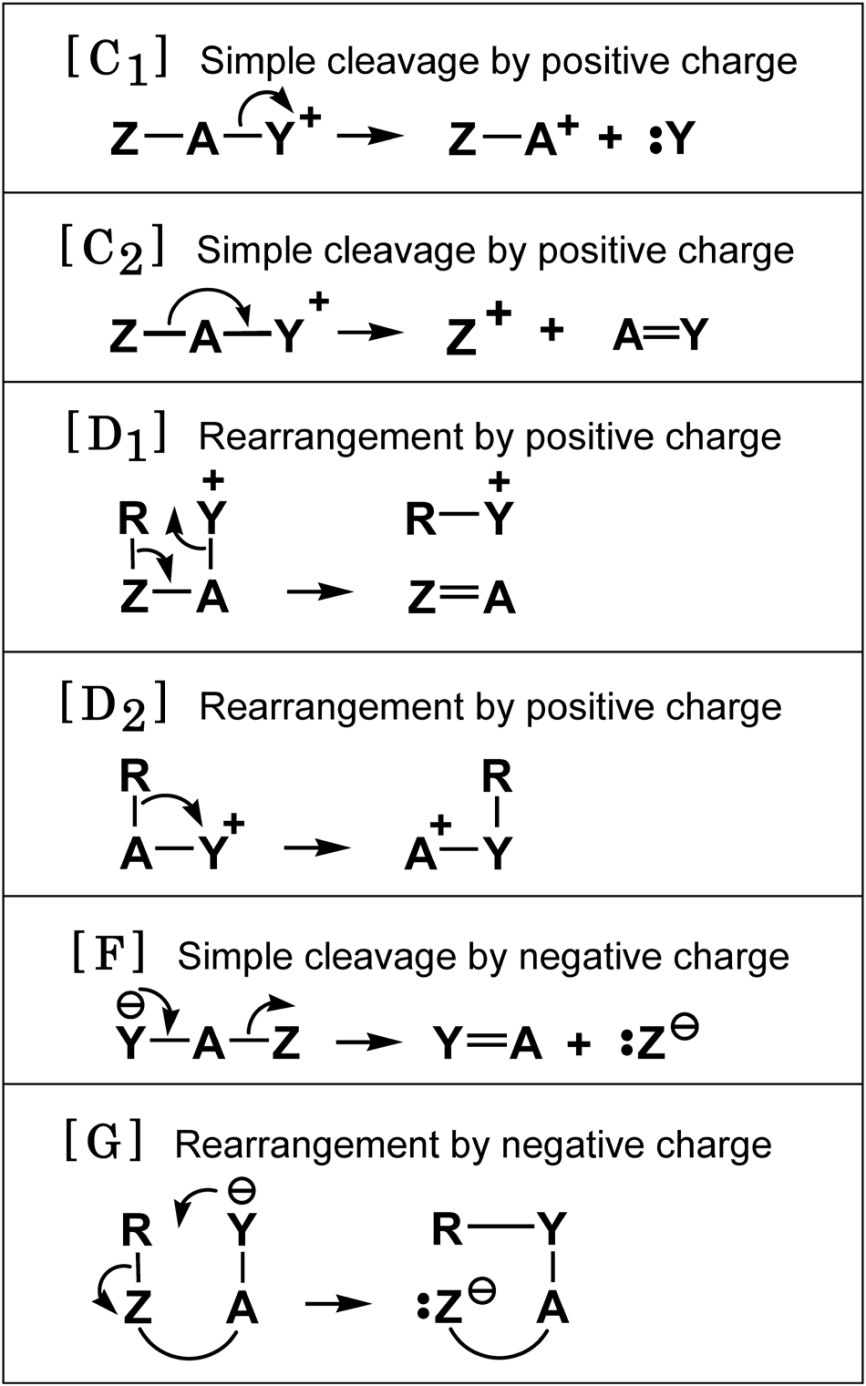

In the case of C_1_, a cleavage occurs when the leaving Y with a formal charge is an electron-rich species with 8 electrons in the outermost shell (such as an ammonium or oxonium ion). It can also occur in cases where Y is an atom with large electronegativity such as Br or Cl (when the unpaired electrons and positive charge localize on the Br or Cl, and the outermost shell of these atoms have only seven electrons). The C_1_ cleavage often occurs when Y forms a stable species (*e.g.*, H_2_O, CO, CH_3_COOH, *etc.*). With regard to the relative ease of cleavage of Y, it is commonly believed that smaller the proton affinity of :Y formed after cleavage, easier the cleavage (Field rule).^9,31,39)^

In contrast to this, cleavage C_2_ occurs on a carbocation, an electron-deficient species, having a formal charge and only six electrons in the outermost shell. Only exception is found in the case of R−C^+^=O, generated from a carbonyl compound *etc.*, where the elimination of carbon monoxide by C_1_ cleavage is preferred over the elimination of a ketene derivative by the C_2_ cleavage. This is because the carbon atom of carbon monoxide (C=O) has an exceptionally stable divalent carbon (carbene).

In addition, if we consider that the above-mentioned C_1_ and C_2_ cleavages are attributed to the attraction of two adjacent bonding electrons by the positive charge on atom Y, it is conceivable that in some cases this attraction works on a single electron as well. We therefore cannot ignore the S_1_ and S_2_ cleavages^9,29,30)^ as shown in Table 3. Although this process violates the Even-electron rule,^31,34,38)^ many such cases have been reported.^40,41)^ In particular, it should be also noted that the Even-electron rule does not necessarily hold when the precursor ion is given a large amount of energy by collisional activation *etc.*

**Table 3:** Table 3. Supplementary bond cleavages by a positive charge.

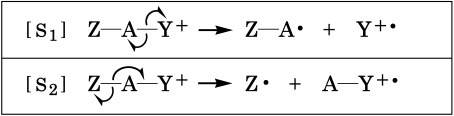

Similar to C_1_, the D_1_ reaction involves a rearrangement cleavage due to a positive charge and occurs in the case of electron-rich ions (*e.g.*, ammonium or oxonium ion). Here again the species Y, having a formal charge, has 8 electrons in the outermost shell, and the R group that rearranges often turns out to be a hydrogen atom. In contrast, the D_2_ rearrangement occurs when Y is an electron-deficient carbocation with a formal charge. This is essentially the same as rearrangement reactions of hydrogen and alkyl groups seen for carbocations in solution. In some cases, electron-rich nucleophilic groups such as phenyl and methoxy groups also rearrange by this mechanism.

Although there are not very many examples of fragmentation from negative ions than those from positive ions, we can formalize the fragmentation modes as F-cleavage and G-rearrangement (Table 3) as only provisional postulation. These fragmentations follow exactly the same pattern as the cleavages A and B_1_ respectively, where the unpaired electron is replaced by the formal negative charge.

## 3. *m*/*z* OF ION OBSERVED AS A PEAK

As is well known, in a typical mass spectrum of an organic compound, the actual data of measurements are *m*/*z* values and relative ion quantity of various ions formed from a given sample in a mass spectrometer. The important challenge in fragmentation analysis is to understand the basis of these two measurements. In order to estimate the *m*/*z* of these ions, we usually assume the structure of the precursor ion and use an appropriate rules of bond cleavage. It is then possible to predict the structure of the fragment ion and calculate its *m*/*z* value. In other words, the calculation of the *m*/*z* of the fragment ion is based on the rules of bond cleavages discussed in Section 2 above.

As shown in Scheme 1, for example, if the structure of the sample compound is simple, prediction of the structure of the precursor ion is simple and clear, and the calculation of *m*/*z* of its fragment ion is also easy because the structure of each fragment ion is depicted by using bond cleavage rules (fragmentation rules) shown in Sections 2.1 and 2.2.

**Figure:**
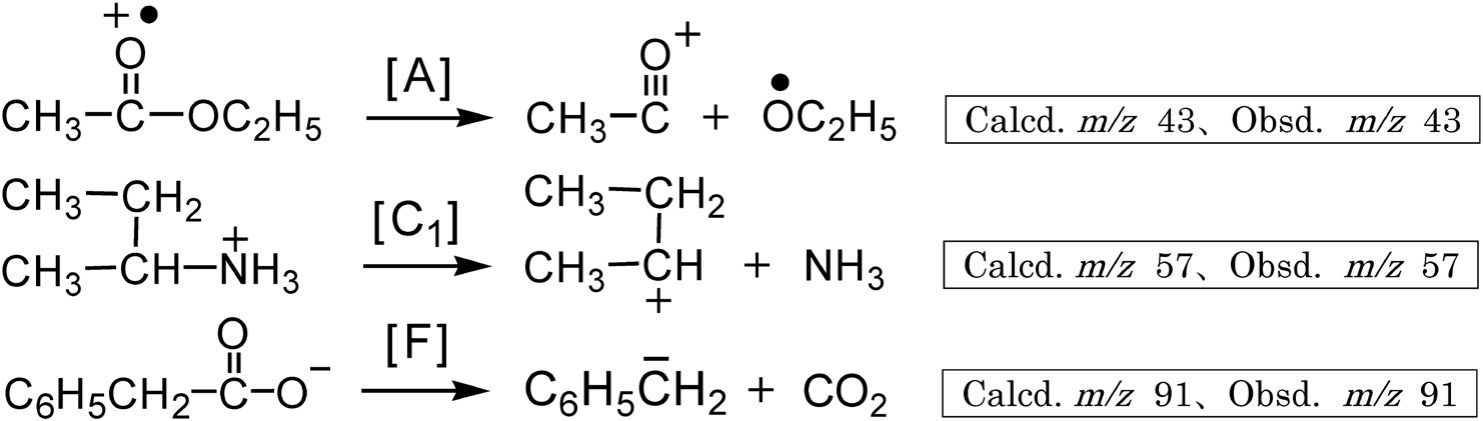
Scheme 1.

A big limitation of these approach is that when the structure of the sample molecule becomes complex, particularly if the sample compound contains multiple heteroatoms (like nitrogen and oxygen), it becomes difficult to make simplified predictions of the position and/or distribution of charge and unpaired electron on the precursor ion. This makes it difficult to apply the rules of bond cleavage to these fragmentations.

As an example, if the structure of the sample is the one shown in Scheme 2, can the bond cleavage and structure of the fragment ion be predicted clearly ? The routine approach to solve this problems is to speculate the position of the cleaved bond of the sample compound from *m*/*z* values in the actually recorded mass spectrum and to estimate the structure of the fragment ions.

**Figure:**
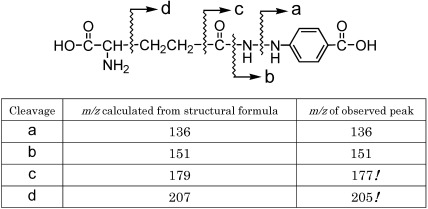
Scheme 2.

In the case of the negative ion mass spectrum of the compound shown in Scheme 2,^42)^ the observed peak at *m*/*z* 136 on the actual spectrum is estimated to have been generated through cleavage at position **a**. The calculated mass of this part of the sample molecule agreed well with the observed *m*/*z* of the peak in the spectrum. Although the reaction in this case appears to be one bond cleavage and seems like very simple, it is not simple to apply the fragmentation rules to this cleavage because it is not clear at which position the negative charge is localized on the precursor ion. In any event, assuming that the cleavage occurs at position **a**, the *m*/*z* of the ion formed is calculated to be 136 from the structural formula, and matched well with the observed peak. Similarly, for the cleavage at position **b** in Scheme 2, the nominal mass calculated from the structural formula matches the *m*/*z* of the peak in the actual spectrum.

On the other hand, a single bond is also supposed to be cleaved at **c** and **d**, but the calculated mass of the corresponding part of the structural formula does not exactly match the observed *m*/*z*, though was close to the *m*/*z* of the peak. Therefore, it could not be said with certainty that the cleavages occurs at these positions. As mentioned earlier, it is not possible to apply the rules of bond cleavage to the structurally complex compounds containing many heteroatoms.

These are particularly serious problem nowadays, when new ionization methods have been developed and spectra of a larger variety of compounds can be measured. Despite the necessity to elucidate the structure of fragment ions corresponding to each peak as well as their mechanism of formation, we have been unable to find a way to verify these problems.

## 4. STRUCTURE OF IONS OBSERVED AS MASS SPECTRAL PEAKS

It will not be an exaggeration to say that, traditionally, no special information on the structure of fragment ions had been provided. Looking at the history of the development of organic mass spectrometry, one can see that while there are many studies related to the structure of individual fragment ions, there has not been systematic studies from a comprehensive point of view. Although there are studies that have examined and shown the most common ion structures that are observed as peaks,^38,43)^ the question why such ions are observed frequently was answered only from an empirical basis. There has been little discussion on the fundamentals of why ions with such structures are often observed. For this reason, the present author attempted to examine the structure of ions that appear as peaks in mass spectra, independently from fragmentation rules discussed above.

During the process of an ion being generated in the mass spectrometer and being observed at the detector, it is important to realize that the ion is stable enough within a fixed lifespan. If a given ion is unstable, it will undergo further bond cleavage to give even smaller ions during the flight pass of a mass spectrometer and is not observed as a normal peak. This is the reason why the present author argues and is focusing on the fundamental aspect of stability of organic ions in the gas phase, with the hope that might shed some light on the problem.

### 4.1 What is a stable ion ?

This brings us to the question as to what would be a criterion or an index to judge the stability of a given ion in the gas phase. Going back to the basics of organic chemistry to revisit the discussions on the stability of normal organic compounds, we can find various indices of stability.^44)^ Some examples include: (1) small internal energy of the compound, (2) small free energy of the compound, (3) facile synthesis of the compound, and (4) poor reactivity of the compound. Conversely, compounds lacking in these properties would be regarded as unstable.

For example, assuming that there are two structural isomers, if we compare magnitude of their standard heat of formation to discuss the stability, the discussion uses index (1) mentioned above. In other words, this is a comparison of the thermochemical stability of these compounds. In the case of cyclobutadiene, one would use index (3) above to say that it is very difficult to synthesize because of its unstable structure. However, considering the application of these criteria to organic ions, while indices (1) and (3) seem feasible, general application would be a very challenging, and it is expected that there will be problems in trying to digitize these criteria as well. We therefore look further back into the basic criteria and try to remember what we learned when we took our first steps into elementary organic chemistry.

What are the basic principles that we follow when drawing the structural formula of a normal organic compound? The basic approach is to draw a structural formula in which each atom involved satisfies the allowed valence state, *e.g.* carbon atom is tetravalent, hydrogen is monovalent, nitrogen is trivalent and oxygen is divalent, and so on. If we draw a structural formula containing divalent carbon atom or monovalent nitrogen atom, the compound with such a structure would not be stable under normal circumstances. In other words, a given organic compound is stable enough to be stored in a reagent bottle in a laboratory if you can draw a structural formula of the compound by using only the normal, allowed atomic valence states of each constituent atom. While there are well-known limitations in representing electron delocalization (*e.g.*, as benzene), organic compounds should be stable if you can write their “structural formulae” as representation of the order of linkage of each atom making up the compound, the types of bonds between each atom (single bond, double bond, *etc.*), and in some situations as depicting the spatial relationships between each atom. Of course, this constraint is a necessary condition for a given compound to be stable and exist in reality, but not a sufficient condition. This means that if you draw the structural formula of a compound according to the aforementioned rules of atomic valence requirement, it does mean that the compound is not always stable.

As for organic ions in the gas phase, it is quite surprising that only little attention has been focused on their stability. Let us now apply the most basic and primitive “stability criteria” described above to organic ions in gas phase. We can summarize the allowed atomic valence states of each atom for stable positive ions in Table 4 and for negative ions in Table 5.

**Table 4:** Table 4. Allowed valence states of common atoms in positive ions.

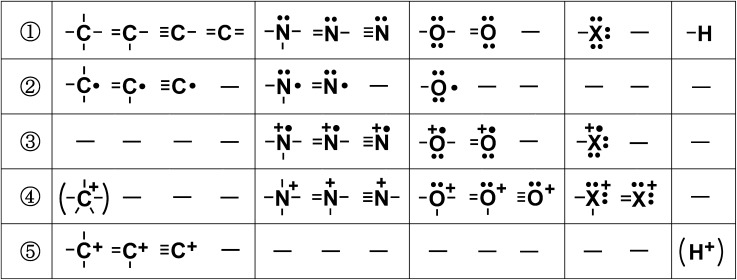

① Valence state in normal organic molecule with no charge and no unpaired electrons ② One valence reduced state with an unpaired electron ③ Valence state with one electron less than the atom’s unshared electron pair ④ Positively charged state with increase of atomic valence (electron sufficient hetero atoms) ⑤ Positively charged state with decrease of atomic valence (electron deficient carbon atom)

**Table 5:** Table 5. Allowed valence states of common atoms in negative ions.

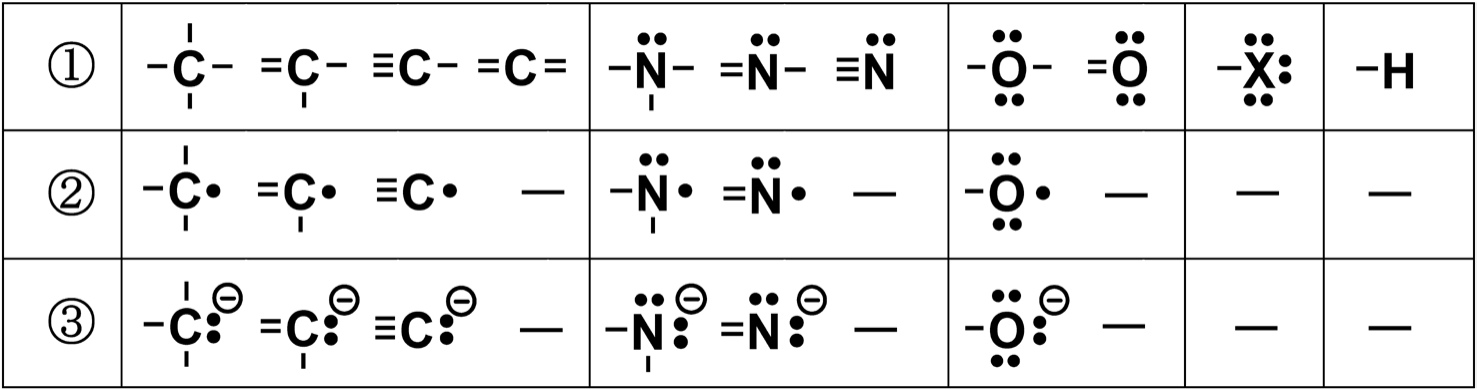

① Valence state in normal organic molecule with no charge and no unpaired electrons ② One valence reduced state with an unpaired electron ③ Negatively charged state with decrease of atomic valence (electron sufficient atoms)

In order for an organic ion to be observed as a peak on the mass spectrum, the ion must be stable, and each atom constituting the ion must be one of the allowed valence states shown in Tables 4 or 5. Any ion with a structure that contains other valence state of atoms than those shown in Tables 4 or 5 cannot exist in a stable form, and the ion with such a structure does not appear as a normal peak on the mass spectrum.

It should be pointed out that in the discussion of the “rule of mass shift” below, as we are dealing with formation of even-electron fragment ions, the valence states of **②** and **③** in Table 4 for positive ions and those of **②** in Table 5 for negative ions should be excluded because these atoms have an unpaired electron and should not be used for constructing “even-electron” ions.

### 4.2 Limitations to the stable structure criterion

Similar to the case of normal organic compounds, we must remember that the aforementioned proposition of ion structure criterion is a necessary but not a sufficient condition for stable ions. If the ion could essentially be drawn by combining atoms of allowed valence states shown in Tables 4 or 5, the ion should be stable enough to appear as a peak on the mass spectrum, but not all structures of ions drawn in such manner will be stable.

For example, the ions with structure shown in Scheme 3 meet the conditions described in Tables 4 or 5, but is nonetheless an example of unstable ions. The reason(s) why such ions are unstable can be conceived in the following manner. Ion [1] has a structure where there is a positive charge next to the carbonyl group, which is a typical electron-withdrawing group, and is therefore unstable due to the electrostatic repulsion of the polarized carbonyl group. Ion [2] is unstable because the conjugated system of cyclic 4 π electrons forms an anti-aromatic system (if an ion with this structure had a negative charge, it would be a stable ion, having 6 π electrons like in a benzene ring). Ion [3] is unstable because the carbon atom with a positive charge is at the bridge-head position, and the structure is unable to form a planar *sp*^2^ trivalent positively charged structure (carbon atom with a negative charge would assume an *sp*^3^ hybridized orbital structure and is therefore not subjected to this limitation). Ion [4] is a terminal carbocation (primary carbocation) of an alkyl chain that does not receive any conjugative stabilization from carbon–carbon double bonds or from the unshared electron pairs of heteroatoms like nitrogen and oxygen, it becomes unstable in the gas phase (it is stable in solution because of the solvent molecules surrounding it to stabilize). Similarly, a primary carbanion [5] at the end of a linear alkyl chain is not stable, unless conjugation from the neighboring double bond. Furthermore, apart from large molecules like peptides, ion with a structure containing two or more positive or negative charges near each other like ion [6], and structures with two or more unpaired electrons situated near each other, are also considered unstable.

**Figure:**
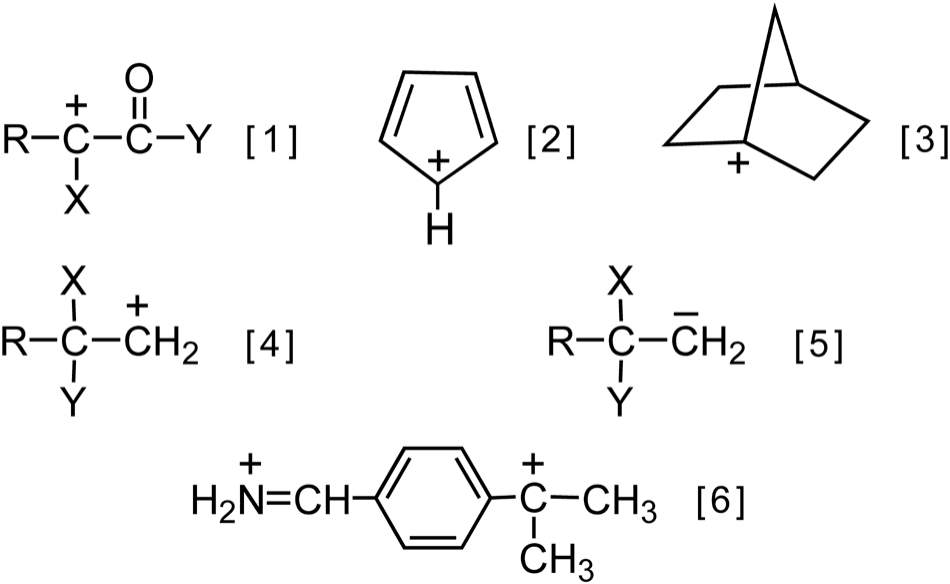
Scheme 3.

## 5. RULE OF MASS SHIFT FOR THE FORMATION OF SINGLY CHARGED EVEN-ELECTRON IONS^2,3)^

Ions generated by EI and CI, as well as selected ions in MS/MS measurement by fast atom bombardment (FAB) or electrospray ionization (ESI), the precursor ions are all isolated in the gas phase. This allows us to apply the rule of bond cleavage for fragmentation described in Section 2 above. On the other hand, with FAB or ESI, where matrices or solvents are used for the effective generation of ions, the actual reaction of bond cleavage might also be affected with the intervention of these environmental materials in the ion source. For this reason, doubts remain as to whether the rule for bond cleavage mentioned in Section 2 can be applied to the fragmentations at the ion source under FAB or ESI ionization, unless we could rule out the possibility that the matrix or solvent is involved in the bond cleavage.

As mentioned in Section 4, an ion observed as a peak in the spectrum is considered to have a stable structure. Therefore, if fragmentation affords an ion of unstable structure, the ion should change to a stable ion by some structural modifications in order to appear as a peak in the spectrum. We define the nominal mass difference due to this structural modification as “mass shift,” and found that the nominal mass (or *m*/*z*, where *z*=1) of a singly charged even-electron fragment ion can be correctly calculated by taking this mass shift value into account (rule of mass shift^2,3)^) (Table 6). The advantage of this procedure is that you just make use of the nominal mass of structural formula of the original molecule itself prior to ionization. You do not need to consider which part a proton is added to or which part a proton is removed from the sample molecule in the ion source. Only thing to do is to assume a specific part of the molecule cleaved.
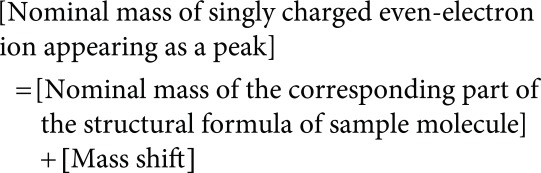


**Table 6:** Table 6. Mass shift for the formation of singly charged even-electron ions.^a)^

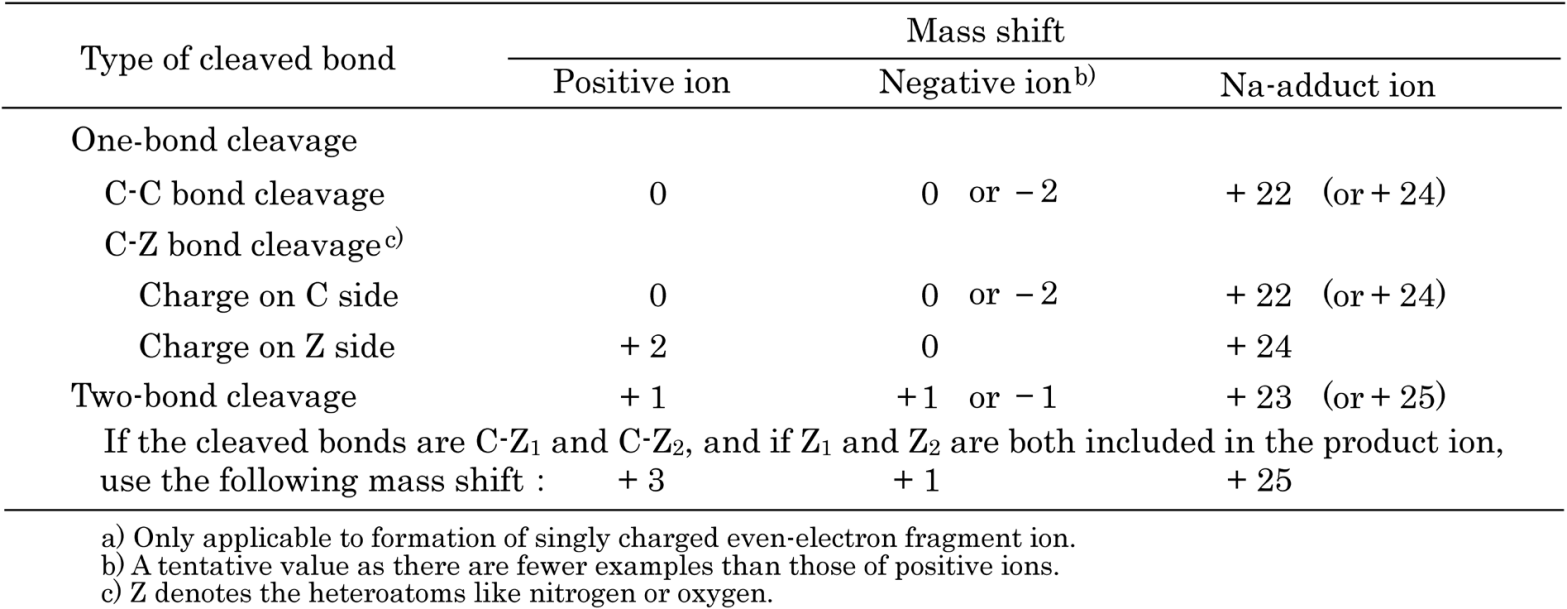

The [mass shift] value in the formula above is determined by three factors, namely (1) positive or negative charge on the ion of interest, (2) number and types of bonds being cleaved, and (3) which side of the two fragments after cleavage will have the charge. Some of the mass shift values for negative ions and Na-adduct ions are revised from originally reported.^2,3)^

### 5.1 Schematic explanation for positive ions

The basic principle of the “rule of mass shift” is that if an unstable ion is formed by bond cleavage from a precursor ion, this unstable ion should be converted to a stable structure by minimal structural changes in order to appear as a peak in the spectrum. Such structural changes for a positively charged fragment ion are schematically shown in Scheme 4, in which the hydrogen atom or proton necessary for stabilization of the resulting ion is denoted by 
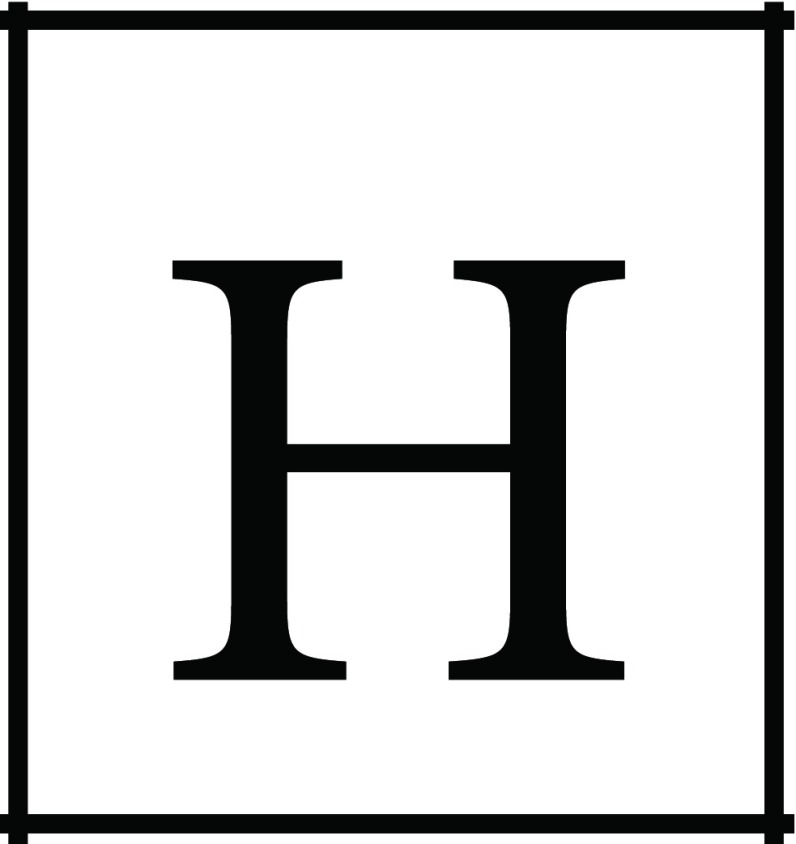
.

**Figure:**
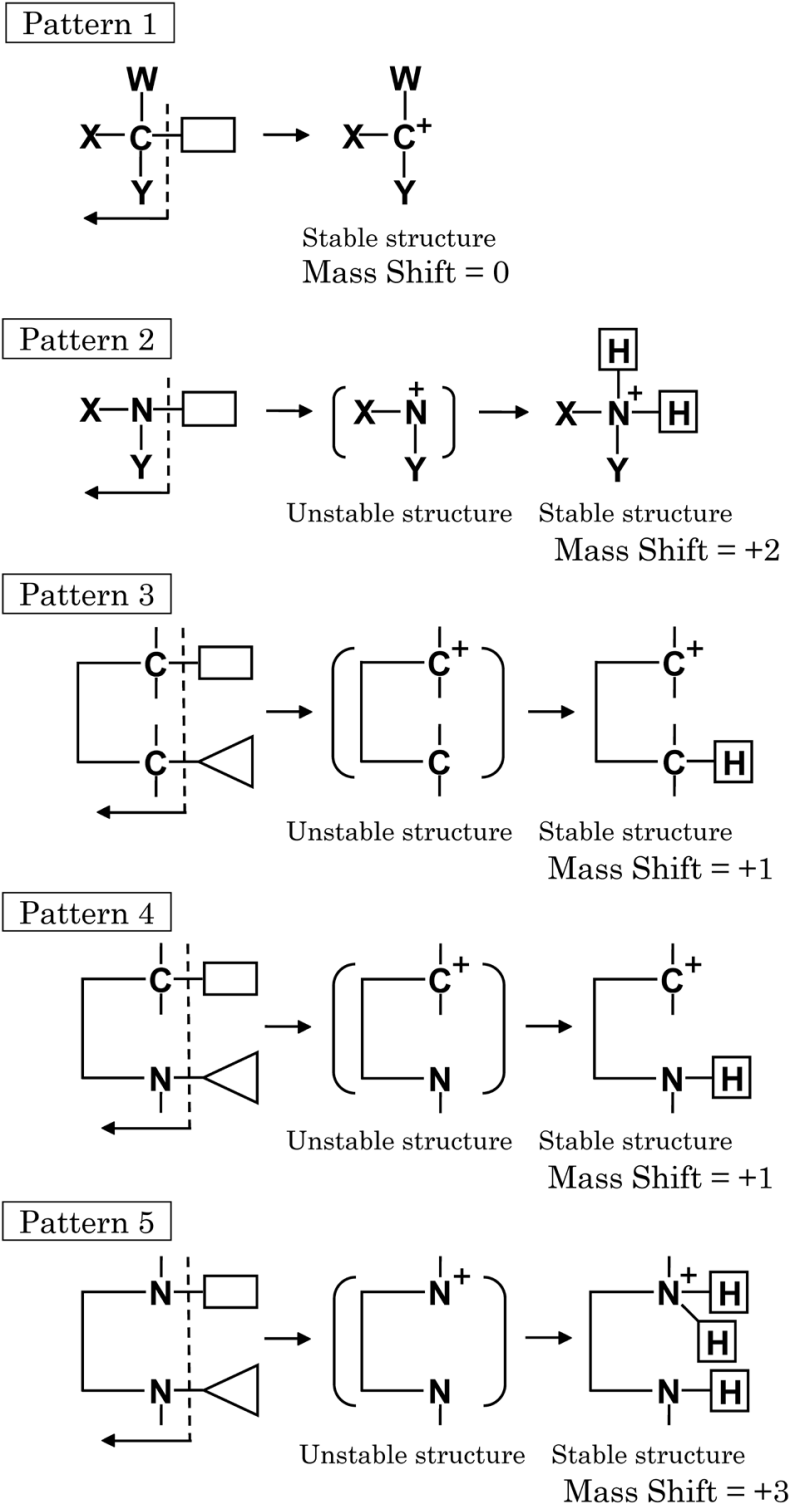
Scheme 4.

**Pattern 1:** In this fragmentation process, when a bond directly attached to a carbon atom cleaves, it creates a positively charged trivalent carbon atom. This situation is not particularly problematic. In other words, since the fragment ion is formed while preserving the composition of that part in the original compound, there is no need for any further structural changes, and the mass shift will be 0 in these situations. However, if the carbon atom ending up with the positive charge after cleavage is in the form of a primary carbocation at the end of an alkyl chain, and without receiving any stabilization *via* conjugation, the structure will be unstable in gas phase as mentioned earlier in Section 4, and it will not be observed in this form. The ion normally stabilized through a D_2_ rearrangement of a neighboring hydrogen atom or alkyl group in order to change the position of the positive charge and form a secondary or tertiary carbocation. There is no change in the *m*/*z* by this rearrangement, and the mass shift also remains unchanged as 0.

**Pattern 2:** Here we consider the situation where a bond between carbon and heteroatom (Z) is cleaved. Z is assumed to be a nitrogen atom, for example. If the fragment ion generated has a positive charge on the carbon atom after the cleavage of the C–N bond, the argument is the same as that presented above for Pattern 1, and consequently the mass shift is 0. On the other hand, if the nitrogen atom ends up with the positive charge, some sort of structural changes must take place, as it is impossible for a nitrogen atom to be in a positively charged divalent state (see Table 4).

As can be seen from Table 4, the only possibility for a nitrogen atom to retain a positive charge and also to be stable, is to convert the nitrogen to a positively charged tetravalent form (ammonium ion). The only way for this to happen is by bonding of two hydrogens to the nitrogen atom and resulting in a mass shift of +2.

It is well-known in the fragmentation of a positively charged peptide chain, that if an acid–amide bond cleaves and leaves a positive charge on the nitrogen side (Y and C cleavage by Biemann representation^27)^) the peak position always shifts by +2 from the value calculated from the original structural formula. This can be considered a typical and well-known example of the mass shift +2 case discussed above.

**Pattern 3:** Pattern 3 shows a situation where two bonds are cleaved simultaneously. If carbon atoms are involved in both bonds being cleaved, and both carbon atoms end up in the fragment ion, one of the carbon atoms is in a positively charged trivalent state, which is an allowed valence state as shown in Table 4. However, the other carbon atom becomes an uncharged trivalent form, which is forbidden valence state, and therefore needs to undergo the least structural changes, where one hydrogen atom binds to it to produce a non-charged tetravalent carbon (mass shift=+1).

**Pattern 4:** When two bonds are cleaved and one of the bonds involves a heteroatom, as seen in Pattern 4, such a heteroatom will not be stable to survive (divalent neutral nitrogen, for example) and will require a hydrogen atom to bind and lead the mass shift of +1. We also arrive at the same result if we assume that after the bond cleavage the free valence of each atom might come together to make a bond (ring formation), and in order for the molecule to have a positive charge, a hydrogen atom (proton) must be added to the heteroatom to afford an onium ion structure, resulting in a mass shift of +1.

**Pattern 5:** This pattern shows an example when both cleaved bonds have heteroatoms and both heteroatoms end up in the fragment ion. As can be imagined from the arguments presented thus far, a minimum requirement for the generated ion to have a stable structure is to bind with 3 hydrogen atoms, resulting in a mass shift of +3.

### 5.2 Schematic explanation for negative ions

Scheme 5 illustrates the formation of negatively charged stable fragment ions.

**Figure:**
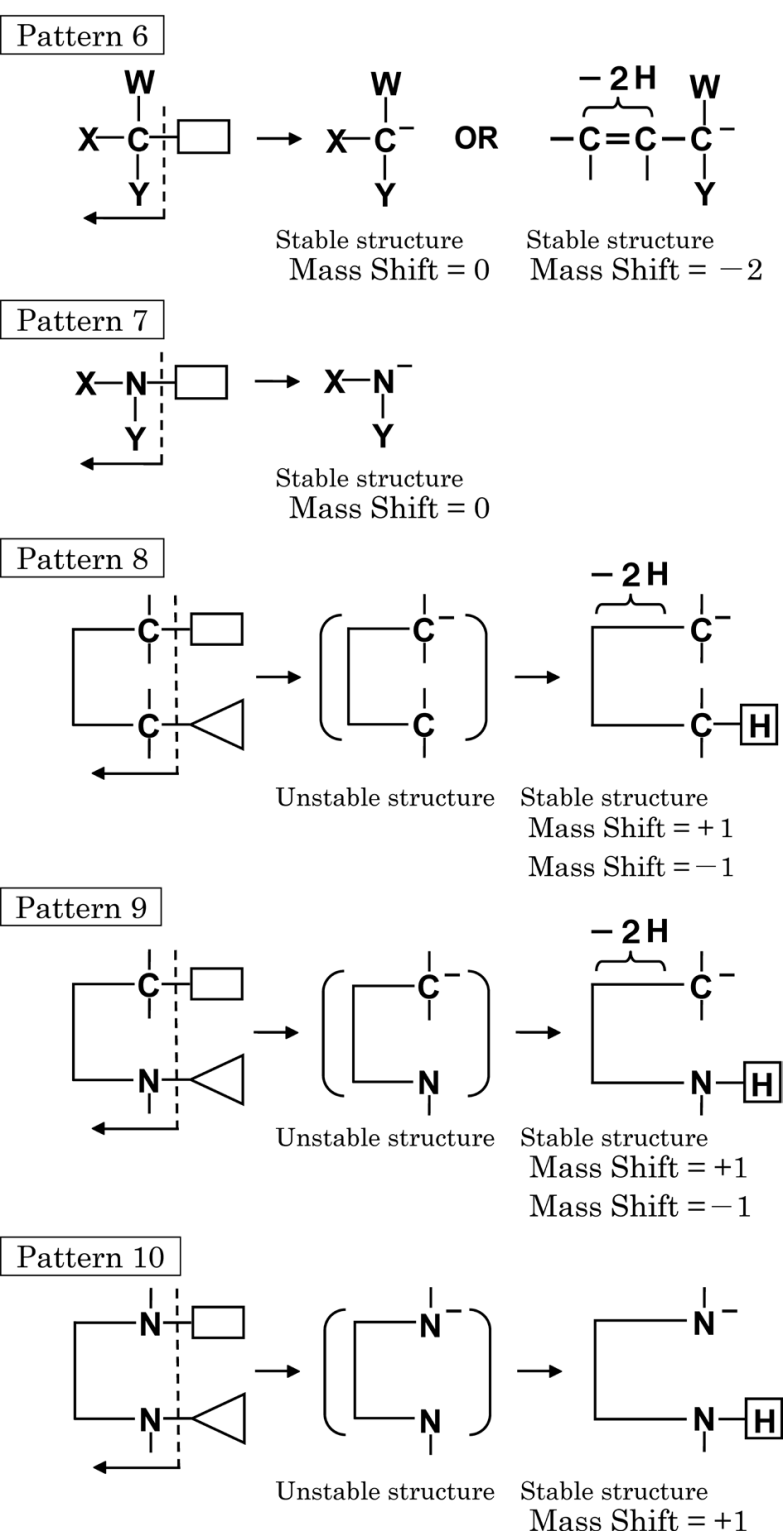
Scheme 5.

**Pattern 6:** This is a fragmentation process where a bond attached to a carbon atom cleaves and leaves negative charge on the carbon atom. The situation is not particularly problematic because the fragment ion has an allowed negatively charged trivalent carbon atom. Under normal circumstances, the mass shift will be 0. However, if the negatively charged carbon atom is at the end of an alkyl chain (*i.e.*, both W and Y are hydrogen), we need to consider two situations. Firstly, if the corresponding carbon atom is stabilized by conjugation from nearby double bonds, or if the entire ion can stabilize through special structural modification, the mass shift remains 0. Secondly, if this primary carbon atom with a negative charge is not able to take part in either conjugation or the special structural modification, it will be unstable in gas phase on the basis of the arguments presented in Section 4, and will not be observed as this structure. Looking at the actual spectra of various compounds, two hydrogen atoms were often removed from the nearby positions to form a carbon–carbon double bond, and subsequent stabilization of the negative charge by conjugation. Hence, the removal of two hydrogen atoms results in a mass shift of −2.

**Pattern 7:** Let us consider the situation where a carbon-heteroatom (Z) is cleaved, Z being a nitrogen atom as an example. If the fragment ion has a negative charge on the carbon atom after cleavage, the argument remains the same as that for Pattern 6, and the resulting mass shift is either 0 or −2. On the other hand, if the negative charge remains on the nitrogen side, a negatively charged divalent nitrogen atom is an allowed valence state and no further structural changes are required for stabilization, and thus the mass shift is equal to 0.

**Pattern 8:** If two bonds are cleaved and atoms involved are both carbon atoms, the mass shift, in general, is +1. If the stabilization of a negatively charged primary carbon atom requires conjugation by removal of two hydrogen atoms, as Pattern 6, the mass shift is +1−2=−1.

**Pattern 9:** If two bonds are cleaved and a negative charge is on the carbon atom in the product ion, the mass shift is +1. If a structural change to form a double bond to stabilize the negative charge through conjugation, the mass shift will be +1−2=−1. Furthermore, in a structure where the heteroatom (nitrogen, for example) ends up with the negative charge, divalent nitrogen atom is allowed valence state and therefore the mass shift is again +1.

**Pattern 10:** If two bonds are cleaved and both bonds involve heteroatoms, the result is a straightforward and the mass shift equal to +1.

### 5.3 Application of the rule of mass shift to precursor ions

The calculation of *m*/*z* for the formation of singly charged even-electron fragment ions in a normal mass spectrum is based on the original structural formula of the compound, prior to ionization. However, when applying the rule of mass shift to the fragmentation of ions, such as the detection of secondary product ions by MS/MS, it is important to note the position of the charge before and after the bond cleavage. If the product ion does not contain the charged part of the precursor ion, the normal mass shift values can be used (Table 6). On the other hand, if the product ion after cleavage contains the charged part of the precursor ion, it is necessary to correct the mass shift values by −1 for a positive ion and +1 for a negative ion (see Scheme 6 and Table 7).

**Figure:**
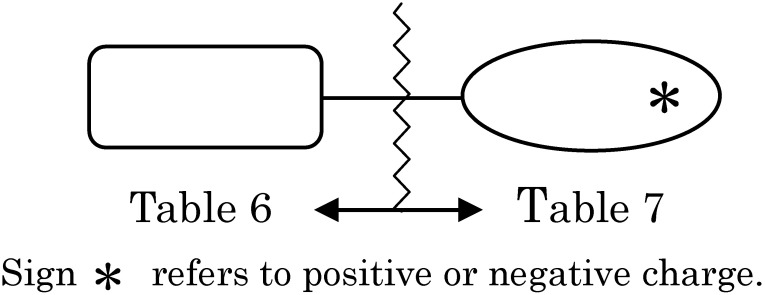
Scheme 6.

**Table 7:** Table 7. Supplementary mass shift for the formation of product ions that retain precursor’s charge site.

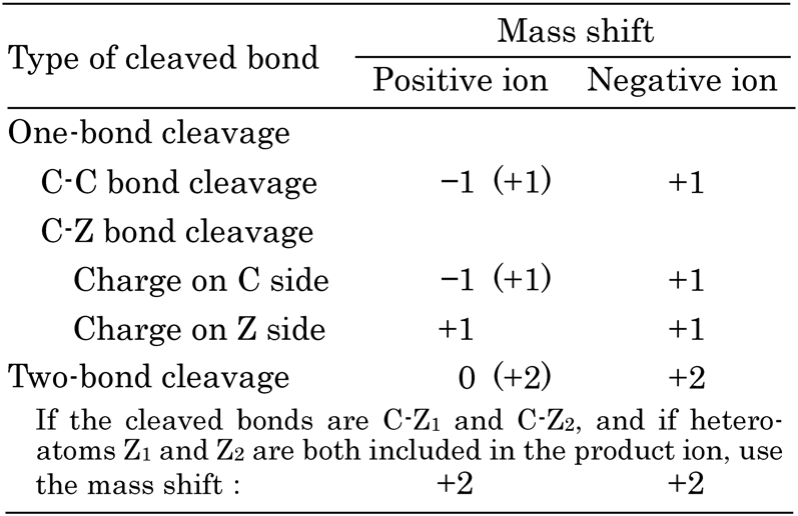

Let us look into more details of the situation where the product ion contains the charged part of the precursor ion by referring to Schemes 4 and 5.

First, in Pattern 1, our prerequisite is that the precursor ion has already a positive charge on one of W, X or Y, and the carbon atom participating the bond cleavage need not to hold a charge after the fragmentation. Therefore, this carbon atom should change to an uncharged and allowed valence state. This requires either elimination of a proton from an adjacent position to form a carbon–carbon double bond, or addition of one hydrogen atom to form an uncharged tetravalent carbon atom. The resulting mass shift will be either −1 or +1. In most cases, the former (mass shift of −1) is prevailing.

In Pattern 2, the positive charge resides on X or Y in the precursor ion and the nitrogen atom in the product ion does not need to hold a charge, the mass shift will be +1 if the nitrogen atom changes to an uncharged trivalent state.

In Patterns 3 and 4, as the positively charged carbon atom in the product ion needs to convert to an uncharged state, the mass shift will be −1 or +1, in a manner similar to Pattern 1. As a hydrogen atom will subsequently bound to the other carbon or nitrogen atom, the final mass shift value will be 0 or +2. It has been seen in many cases that the mass shift tends to be 0.

In Pattern 5, as both heteroatoms that are directly involved in the cleavage (nitrogen atoms in this particular example), a change to an uncharged state will result the mass shift of +2.

In the case of negative ions, if the product ion contains the charged part of the precursor ion, the result will be as follows. First, the argument for Pattern 6 in Scheme 5 is same as that for Pattern 1 with positive ions. As the negative charge will be localized on one of W, X or Y, the carbon atom at the site of bond cleavage does not need to hold the negative charge, and the carbon atom simply needs to bind with a hydrogen atom to become an uncharged tetravalent carbon atom. The mass shift therefore is +1. As there is no need to stabilize the negative charge, no additional structural change will occur such as releasing two hydrogen atoms to form a carbon–carbon double bond.

In Pattern 7, as the heteroatom (nitrogen atom in this example) directly involved in the bond cleavage needs to be an uncharged state, bonding with one hydrogen atom will result in the mass shift of +1. In Patterns 8 and 9, both carbon and heteroatoms after the cleavage need to change to uncharged states. Hence, the mass shift will be +2 in both cases. Furthermore, as with Pattern 6, there is no need to remove two hydrogen atoms to stabilize the negative charge by conjugation. In Pattern 10, as both cleaved heteroatoms need to be in a stable atomic valence state with no charge, the mass shift can be easily determined to be +2.

For making the calculation of *m*/*z* easier and simpler, we depicted the two-bond-cleavage reaction as Patterns 3, 4, 5 for positive ions and Patterns 8, 9, 10 for negative ions as above. However, the simultaneous cleavage of two bonds is not very common in mass spectral fragmentations. Therefore, it may be more realistic to think that the cleavage of these two bonds takes place successively in two steps. Since the precursor ion for the second step (product ion from the first step) already has a charge, the product ion of the second step contains the charged part of the precursor ion and thus the mass shift values of Table 7 should be used.

A special feature of the rule of mass shift, as mentioned earlier, is that the calculation to predict the *m*/*z* of observed peaks can be carried out on the structural formula of the sample compound itself, prior to ionization. One of the advantages is that there is no need to think about where protons are added or removed during the ionization step in ion source. However, depending on the structure of the sample molecule or on the method of ionization, it may be possible to predict clearly the protonated or deprotonated position on the sample molecule. If the rule of mass shift is applied to such cases, one may judge whether or not the product ion contains the charged part of the precursor ion, and decide whether to use Table 6 or 7 to calculate *m*/*z* of the product ion. This has been described using several specific examples in a previous review.^3)^ The approach provides exactly the same results with no exceptions as those obtained from calculations based on the original, charge-free structural formula of the compound prior to ionization.

One more advantage of the above rule of mass shift for calculation of *m*/*z* of fragment ions is that the method is also applicable to monovalent “odd-electron” molecular ions of organic compounds. Only necessary requirement for the rule to be valid is the case of singly charged even-electron “fragment ions,” and accordingly if odd-electron molecular ions undergo bond cleavage reaction to afford singly charged even-electron fragment ions, the rule can correctly estimate their *m*/*z* values.

## 6. DEVELOPMENT OF THE RULE OF MASS SHIFT AND FUTURE CHALLENGES

Around the time when people started to measure mass spectra of organic compounds, our understanding of fragmentation of organic molecules was still under twilight, and various wavy-line notations were used to discuss bond cleavages. It was a simple approach that one may cut up the structural formula of a sample molecule into a specific part of segments by using wavy lines and compare its nominal mass with *m*/*z* that would be observed, assuming that the molecule would fragment to give ions of that particular part (*e.g.*, Scheme 7). Analysis of the substantial amount of data acquired thereafter led to the construction of fragmentation rules described in Section 2, which became more and more organized. Now it is not too much to say that we have reached a sophisticated stage of understanding bond cleavage of organic compounds in gas phase, particularly on the fragmentations by EI or CI.

**Figure:**
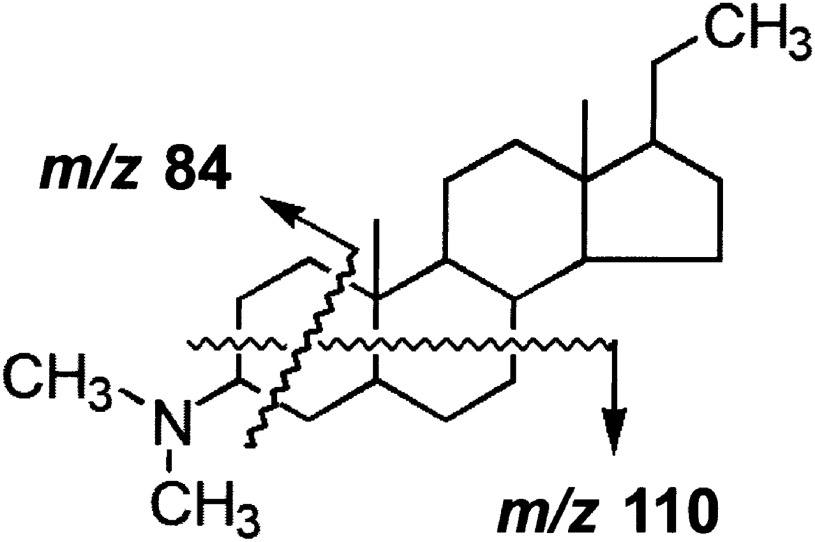
Scheme 7.

On the other hand, fragmentations caused by FAB or ESI involve much more complex phenomena with many unanswered problems. In terms of bond cleavage, we are only at a primitive and rudimentary stage of understanding. Although application of the “rule of mass shift” was first reported to be useful in the prediction of *m*/*z* of singly charged even-electron fragment ions generated by FAB ionization, examination of various data accumulated later showed that at least for monovalent even-electron ions, the rule can correctly predict the *m*/*z* of fragment ions by ESI, MALDI (matrix-assisted laser-desorption ionization) and charge-remote fragmentations as well, for which the modes of bond cleavage have not yet been established. As several practical examples were already explained in detail in a previous article,^3)^ we will limit the description to two other characteristic cases below.

First, Scheme 8 shows the cleavage of the protonated molecule obtained by the ESI of a metabolite of a particular compound (C_24_H_29_N_3_O_10_S, nominal mass: 551).^45)^ Table 8 shows the results from applying the rule of mass shift to each cleavage. We can see that the *m*/*z* values of the ions formed by cleavages from (a) to (f) have been correctly predicted. While the predicted *m*/*z* for the cleavages at (a′) and (f′) are the same as those for the cleavages at (a) and (f), the composition of ions differ respectively for these two sets of ions. It would be instructive to recheck this with the help of a high resolution mass spectrum.

**Figure:**
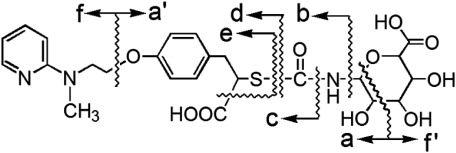
Scheme 8.

**Table 8:** Table 8. Product ions from a protonated precursor ion.

Cleavage	Composition of ions	Nominal mass	Mass shift	Observed *m*/*z*
a	C_20_H_23_N_3_O_5_S	**417**	+**1**	**418**
a′	C_16_H_18_NO_10_S	**416**	+**2**	**418**
b	C_18_H_20_N_3_O_4_S	**374**	+**2**	**376**
c	C_18_H_19_N_2_O_4_S	**359**	**0**	**359**
d	C_17_H_19_N_2_O_3_S	**331**	+**2**	**333**
e	C_16_H_18_N_2_OS	**286**	+**1**	**287**
f	C_8_H_11_N_2_	**135**	**0**	**135**
f′	C_4_H_6_O_5_	**134**	+**1**	**135**

As the second case, Scheme 9 shows an example where the rule can be applied effectively even though the ionization method used is MALDI. Data shown in Table 9 are obtained from high resolution MS/MS spectrum and fragmentations are from a sodium-ion adduct molecule of lactosylceramide C_54_H_103_NO_14_+Na^+^. The main product ions are formed by cleavage at positions shown on Scheme 9, producing five peaks.^46)^

**Figure:**
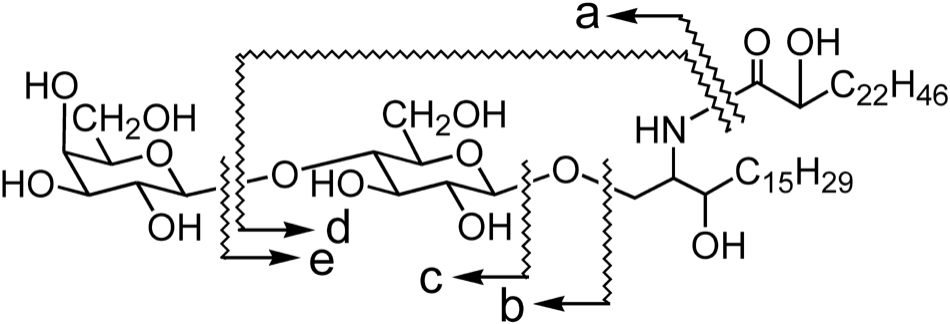
Scheme 9.

**Table 9:** Table 9. Calculated and observed *m*/*z*.

Cleavage	Calculated value	Mass shift	Predicted *m*/*z*	Observed *m*/*z*
a	622.3787	+23.9976	**646.3763**	**646.4**
b	341.1077	+23.9976	**365.1053**	**365.1**
c	325.1128	+21.9820	**347.0948**	**347.1**
d	459.3184	+25.0054	**484.3238**	**484.3**
e	826.6748	+23.9976	**850.6724**	**850.7**

When applying the rule of mass shift to high-resolution mass spectrum, as in this example, the mass shift value should be calculated using non-integers but exact masses, such as 1.0078 for the hydrogen atom and 22.9898 instead of 23 for the sodium ion. As can be seen clearly from Table 9, the predicted *m*/*z* and the measured data agree very well.

It is important to realize that not all fragment ions generated from a sodium ion-adduct molecule contain sodium. Namely, fragment ions that do not contain sodium may also be formed. To apply the rule of mass shift to such sodium-free fragment ions, mass shift values for normal ions (Table 6) should be used. Conversely, by using which mass shift values match the *m*/*z* of the actually observed peaks, we are able to differentiate whether the fragment ion from sodium-adduct ion contains sodium or not. A commentary on two practical examples have been provided earlier.^3)^

As discussed in greater detail above, the basic idea regarding the rationale for establishing the rule of mass shift is that the fragment ions observed as peaks on a normal mass spectrum must have stable structures. After bond cleavage, any fragment ions that contain atoms of unstable forbidden valence states need to change to allowed valence states as shown in Tables 4 or 5. Usually a specific number of hydrogen atoms (or a proton) can be added or removed to achieve these structural modifications that are schematically depicted as Scheme 4 or 5. Presently, however, no explanation can be provided for either the origin of the hydrogen atoms (or a proton) that bound to the product ion or the mechanism of such structural changes. Although it is conceivable that the matrix or solvent are actively involved in these processes for FAB and ESI, we still have no experimental evidence to support this notion. On the other hand, as the rule of mass shift is also valid for MS/MS and charge-remote fragmentations, where the fragmentation occurs from isolated ions in gas phase, it is easily expected that hydrogen-atom transfers take place from the uncharged neutral partner of the fragmentation *via* ion-neutral complexes. We look forward to future studies in this direction to further clarifying our understanding.

## 7. CONTRIBUTION TO FRAGMENTATION ANALYSIS

In order to understand the fragmentation of organic compounds, the first challenge is to elucidate the structure of ions corresponding to each peak on the measured mass spectrum as well as the mechanism of their fragmentation reactions. For this purpose, after estimating the position of unpaired electron and positive or negative charge on the precursor ion, fragmentation rules were applied to deduce the bond changes taking place during the fragmentation. As is well-known, in the case of fragmentation from EI and CI, the precursor ion is in an isolated state in the gas phase, and these approaches work sufficiently well at least for simple compounds and have produced many fruitful results.

However, once the structure of the sample compound becomes complex or contains a number of heteroatoms, it is not easy to answer even basic questions like from which part an electron is removed to produce the molecular ion, or where the proton is added to or taken from the sample molecule to give the protonated or deprotonated ion. As a result it also becomes difficult to apply the rules of bond cleavage in these cases to discuss fragmentation mechanisms. Things become even more complicated for FAB ionization as well as for ESI and MALDI methods, where the mechanism of ionization itself is yet to be fully understood due to the possible intervention of matrix or solvent during the ionization. Therefore, even the rule of bond cleavage cannot be applied. Moreover, it has become increasingly common in recent times to apply energy on ions to induce fragmentation and obtain various kinds of information about the sample compound. In these cases, various types of reactions, such as the charge-remote fragmentation, take place that are apparently unrelated to the fragmentation rules discussed above. Consequently, only by using these rules, it has been very difficult to analyze even basic information about fragmentation, such as the manner of bond cleavages and the structure of the generated fragment ions.

By recognition of the fact that the ion observed as a peak in mass spectrometry must be stable, we proposed the “rule of mass shift,” based on the idea of assessing the stability of the structure of organic ions from structural point of view, in order to aim to accurately predict the *m*/*z* of peaks observed in mass spectra. At an earlier stage of these works, mass spectra of a large number of compounds with known structures were examined to compare *m*/*z* of the peak actually appearing in the spectrum and the nominal mass calculated from the structural formula to determine and adjust the mass shift values. Therefore, the numerical mass shift values can also be considered as statistically meaningful numbers in a certain sense. As already mentioned in Section 5, we finally adopted the following three factors: (1) positive/negative charge on the ion of interest, (2) number and types of bonds being cleaved, and (3) which side of the two fragments after cleavage retains the charge.

However, based on the discussions described in Section 5, we were able to advance the debate to the level of specific structural changes corresponding to the mass shift, and to estimate the structural rationale determining the mass shift. In other words, if fragmentation resulted in the generation of an ion of unstable structure, such ion would undergo some structural changes to transform itself into an ion of stable structure, and is becoming measurable on the mass spectrum. This means that the rule of mass shift not only predicts accurately the *m*/*z* of peaks appearing on the spectrum, but also suggests the structure of the corresponding fragment ions. We can consider this as one of the significant piece of information towards understanding the mechanism of the fragmentation.

Since the rule of mass shift predicts the *m*/*z* of peaks on the spectrum and clarifies which part of the sample molecule has been cleaved, and moreover, presumes the structure of fragment ions, we are now at the stage that we can analyze fragmentations in greater detail than before. It is the time to apply the fragmentation rules of bond cleavages mentioned in Section 2. Even if the sample compound has a complex structure with many heteroatoms, it would be easy to apply the rules of bond cleavage if we were able to identify the bond cleavage sites from the *m*/*z* of the peaks appearing on the spectrum. In this context, the rule of mass shift plays a significant role not only for analyzing the spectrum but also for understanding the fragmentation mechanism.

It is necessary to mention that the discussion of the fragmentation in this manner does not go beyond the scope of estimation, and that the fragmentation can be understood in light of the various rules that have been proposed so far. It is of course necessary to obtain independent experimental proof to establish entire picture of the fragmentation in the near future.

## 8. CONCLUSION

As far as the structure of the sample compound or the precursor ion is known, the “rule of mass shift” is capable of accurately predicting the *m*/*z* of a singly charged even-electron fragment ion by assuming that a specific bond of the sample compound or of the precursor ion was cleaved, irrespective of the ionization method used. Although its application is limited to singly charged even-electron fragment ions, the rule applies without exceptions within that scope of applicability.

Whether the rule of mass shift is applicable to the formation of odd-electron fragment ions with unpaired electrons is currently under study and yet no conclusion has been reached. The issue regarding the rule’s applicability to fragmentation of multiply-charged ions generated by ESI is yet to be addressed as well. Furthermore, as normal prediction of *m*/*z* is based on the structural formula of the sample compound, the application to fragmentations involving large-scale skeletal rearrangements remain a challenge for the future. In addition, the detailed mechanism of structural changes to form stable ions (addition of hydrogen atoms and protons, removal of two hydrogen atoms, *etc.*), which is the basis of establishing the mass shift values, has not been resolved. From these points of view, the rule of mass shift still suffers from several unsolved problems. From the standpoint of fragmentation chemistry, we must realize that the level of development is several steps lower than the established fragmentation rules mentioned in Section 2.

However, since the rule of mass shift is based on the stable organic ion structure, and it is based on the basic principle of organic chemistry called atomic valence structural formula, the rule must hold as long as this basic principle does not break down. In other words, as long as this major principle, which has been fostered and established over the long history of organic chemistry, is not destroyed, we will not see a day when the rule of mass shift will become invalid. In fact, until now, there has been no report stating the invalidity of the rule, as far as the generation of singly charged even-electron fragment ions are concerned.

In spite of the unresolved issues, the rule of mass shift will not lose its usefulness in its scope of applicability when singly charged even-electron fragment ions are formed in spectra by using various ionization methods like EI, CI, as well as FAB ionization, ESI and MALDI *etc*. The rule is also applicable to ions formed by collisional activation such as charge-remote fragmentations. We hope future studies will develop the rule of mass shift further and make significant contributions to the field of fragmentation analysis, along with the rule of bond cleavages mentioned in Section 2.

## Notes

Mass Spectrom (Tokyo) 2019; 8(1): A0074

## Notes

This article is a secondary publication of *J. Mass Spectrom. Soc. Jpn.* 63: 31−43, 2015 (Japanese).
